# The Evolving Role of Ophthalmology Clinics in Screening for Early Alzheimer’s Disease: A Review

**DOI:** 10.3390/vision4040046

**Published:** 2020-10-29

**Authors:** Paris Dickens, Kanna Ramaesh

**Affiliations:** 1 Avon and Wiltshire Mental Health Partnership NHS Trust, Petherton Resource Centre, 3 Petherton Road, Hengrove, Bristol BS14 9BP, UK; paris.dickens@nhs.net; 2 Tennent Institute of Ophthalmology, Gartnavel General Hospital, 1053 Great Western Road, Glasgow G12 0YN, UK

**Keywords:** Alzheimer’s disease, screening, cataracts, eye, dementia

## Abstract

Alzheimer’s disease (AD) is the leading cause of dementia, which is a growing public health concern. Although there is no curative treatment for established AD, early recognition and modification of the known risk factors can reduce both severity and the rate of progression. Currently, an early diagnosis of AD is rarely achieved, as there is no screening for AD. The cognitive decline in AD is gradual and often goes unnoticed by patients and caregivers, resulting in patients presenting at later stages of the disease. Primary care physicians (general practitioners in the UK) can administer a battery of tests for patients presenting with memory problems and cognitive impairment, however final diagnosis of AD is usually made by specialised tertiary level clinics. Recent studies suggest that in AD, visuospatial difficulties develop prior to the development of memory problems and screening for visuospatial difficulties may offer a tool to screen for early stage AD. AD and cataracts share common risk and predisposing factors, and the stage of cataract presentation for intervention has shifted dramatically with early cataract referral and surgical intervention becoming the norm. This presentation offers an ideal opportunity to administer a screening test for AD, and visuospatial tools can be administered at post-operative visits by eye clinics. Abnormal findings can be communicated to primary care physicians for further follow up and assessment, or possible interventions which modify risk factors such as diabetes, hypertension and obesity can be undertaken. We propose that eye clinics and ophthalmology facilities have a role to play in the early diagnosis of AD and reducing the burdens arising from severe dementia.

## 1. Overview of Alzheimer’s Disease 

The most common form of dementia, Alzheimer’s disease (AD) is a progressive and debilitating condition. It is a growing public health concern nationally and globally as life expectancies increase in the UK and the wider developed world. 1.6 million people are predicted to have AD by 2040 in the UK alone while by 2050 there are expected to be 115 million cases of dementia worldwide, with AD accounting for between 60% and 70% of these [1,2]. Studies have identified that AD and cardiovascular diseases share common risk factors, which include diabetes, hypertension, obesity, smoking, physical inactivity and poor dietary habits [3,4,5]. Non-modifiable risk factors in AD include female gender and age, with age also affecting life expectancy in AD sufferers, as people who are diagnosed in their sixties and seventies may live between seven and ten years, whereas people in the nineties are only expected to live for three years.

Two major factors that contribute to AD’s pathogenesis are excessive formation of soluble and insoluble amyloid beta aggregates and deposition of intracellular neurofibrillary tau protein tangles [6,7,8]. The general progression of the disease begins with soluble amyloid beta accumulation and insoluble amyloid deposition. The accumulation of these deposits is attributed to neuronal degeneration and synaptic dysfunction. Emerging evidence supports the theory that deranged intracellular protein metabolism of neurons may underpin the mechanism of neuronal degeneration. Intracellular proteins are constantly renewed, and misfolded proteins are eliminated by the ubiquitin-proteasome system. Faults in the ubiquitin–protease system due to genetic mutations and variabilities allows accumulation of abnormal intracellular proteins, and this has been proposed as a prime driver in the pathogenic mechanism of AD [9]. Eventually there can be a 50% loss of synapses and neurons in the hippocampus, and memory impairments develop. As the disease progresses, synaptic impairment and network disturbances cause wider cognitive dysfunction. In the early stages, short-term memory loss and visuospatial impairment is often present; over time this often causes disorientation, self-neglect, changes in behaviour and increased wandering and restlessness. As the disease progresses, debilitating symptoms including aphasia, lexical anomia, agnosia and executive function may develop [6].

At present, there is no curative treatment for AD. Acetylcholinesterase inhibitors (Donepezil, Rivastigmine, and Galantamine) and NMDA-receptor partial agonists (Memantine) may offer relief of some symptoms, however these therapies are not associated with gain in cognitive function [10,11]. As the current drugs show limited clinical benefits, several interventional studies have shifted focus to identifying at risk individuals of AD and advocating preventive measures to manage AD long term [10,11]. Given AD’s multifactorial nature, it has been suggested that multidomain interventions could be an original and potentially effective way to deal with symptoms arising from AD [5,12].

## 2. Evidence for Benefits of Early Diagnosis of AD

Early diagnosis of AD may have significant benefits for patients, caregivers, healthcare professionals and society as a whole. For patients and caregivers, an early diagnosis may reduce uncertainty and anxiety, improve relationships and quality of life, offer the opportunity to come to terms with the diagnosis and seek support [13]. It may additionally help to avoid crises and is overall extremely relevant to the wellbeing of the individuals [14]. Studies indicate that AD patients generally prefer an early diagnosis and wish for diagnosis to be disclosed to themselves and relatives who may be affected by this news [15,16]. An early diagnosis while mental faculties are relatively intact will allow individuals to make decisions for their future and plan ahead before their capacities decline and facilitate better preparation from families and carers [17].

For healthcare professionals, improved diagnostic pathways may help to avoid “medical nomadism” which can be experienced during the diagnostic process, wherein patients present to various doctors with the same complaint over a short period of time [18]. For services, better workload planning and resource allocation were highlighted in a study that asked primary care practitioners (nurses and GPs), and there may be an increased ability to anticipate future demand [19]. A recent study also speculated that timely diagnosis may avoid prescription of medications that could worsen cognitive function, and suggested that newer and novel disease modifying therapies are likely to be lot more effective if they are initiated early in the course of the AD disease [15].

Institutionalisation of AD patients accounts for a large proportion of the total care costs of AD, and patients assessed and treated early remain in the community longer, reducing this financial burden [19]. A recent literature review examined studies and models that addressed economic benefits of early diagnosis and treatment, concluding that the cost of early diagnosis and early intervention will be offset by subsequent savings achieved from reduction in institutionalisation [15]. Although patients may still require admission to a residential or nursing home in the terminal phase of their disease, the overall length of time in care and therefore cost, would be reduced. A cost-benefit analysis performed in the U.S.A. indicated that net fiscal benefits may be optimised when patients receive an early diagnosis of AD and can consequently benefit from interventions such early pharmacological therapy, and respite for carers [20]. One study estimated that a theoretical reduction of 6%, 10%, and 20% in residential care home admission over the course of ten years would result in savings to UK society of approximately £150 million, £245 million, and £490 million, respectively [21,22]. In summation, this information suggests that the development of more tools and clinical pathways to improve early diagnosis could reduce the financial burden of AD.

## 3. Current Screening and Assessment for Dementia—A Brief Overview

Presently there is no mass screening for dementia, and at the primary healthcare level screening and assessment is undertaken by a general practitioner (GP). GPs may choose various tools depending on resources and time available with the patient. Assessment commonly begins with the “one-minute test”, which is a short conversation about memory with the patient, and is intended to illuminate a need for further, more detailed assessments. Another option is the “Mini-Cog test”, which requires a few minutes. Here patients are asked to memorise a short list of words, draw a clock face and then repeat the memorised words. If a full ten-minute consultation is available, the GPCOG (the General Practitioner Assessment of Cognition) is the most used measure, which is easy to administer and takes in the views of the carer or relative. Finally, a more extensive tool is the Montreal Cognitive Assessment (MOCA), which requires a fifteen-minute planned interview. This assesses different cognitive domains: attention, concentration, executive functions, memory, language, conceptual thinking, calculations, visuospatial skills and orientation. A single consultation may not suffice to make a diagnosis of dementia, and it is recommended to use cognition tools alongside a careful history, discussions with carers and relatives, examination and normal screening blood tests. This commonly allows diagnosis to be made in patients with a typical presentation of AD. If the presentation is more complex, referrals need to be made to specialist clinics such as memory or neurology clinics. Currently there are no extensive visuospatial tests administered by GPs in primary care for the purpose of dementia screening.

## 4. Visuospatial Function in Early AD

Broadly defined, visuospatial function requires identification, integration and analysis of space, in addition to processing visual form, details, structure and spatial relations [21]. It is commonly conceptualised in three components, visual perception, construction and visual memory [23].

Visuospatial skills are the use of vision in the perception of the objects in an environment, and the spatial relationships between them. Integration of visual information occurs via two processing streams that are distinct neural circuits, which project from the striate cortex to the posterior parietal (dorsal) or inferotemporal (ventral) cortices. The dorsal regions process space-based “where” information, while the ventral regions process object-based “what” information [23,24]. Visuospatial dysfunction is thought to be among the earliest manifestations of AD and eventually goes on to affect between 20–43% of patients. It manifests in a variety of impairments including dorsal stream functions like angle discrimination and motion perception, in addition to ventral stream functions such as facial discrimination and recognition of objects and colours [23,25]. Disturbances of functions like reading, visuospatial orientation and visual search strategies may also occur [24]. Mild impairment in this domain has been shown to be a strong predictor of progression to AD, and it has been suggested that a decline in visuospatial function may be present about five to six years before AD is diagnosed [26,27]. Visuospatial function may therefore be of potential use as a cognitive marker for the detection of AD before it has reached a clinical stage or setting [23,28]

## 5. Testing Visuospatial Function in AD Patients

### 5.1. Overview

Visuospatial function is commonly assessed by specific neuropsychological measures, which are necessary as tests of visual acuity and visual field alone are poor predictors of complex vision-dependent tasks that are likely to be impaired in patients with early stage AD [29]. These measures commonly require a fusion of cognitive abilities and a physical motor skills component, which can increase the complexity of interpreting performance. It is therefore crucial to select the most appropriate tests to minimise complexity of interpretation while adequately testing visuospatial function.

### 5.2. Visual Object and Space Perception (VOSP) Battery

Warrington et al. developed and validated the Visual Object and Space Perception (VOSP) battery, which evaluates space and object perception in a manner that demands only simple responses of the participant and minimises the involvement or interference of other cognitive skills. It presumes that object perception and visual perception are independent of one another. In a sample of healthy older adults, this two-factor model of visuospatial perception as proposed by the VOSP has been shown to be accurate using a confirmatory factor analysis approach, while the VOSP has shown good accuracy and good correlation with tests measuring visuospatial function [28,29]. The VOSP has been utilised across differing populations with success and shown no significant differences between non-pathological Spanish, American and English populations [30]. Age was shown to have no effect on performance in a sample of people aged 18–49 [31]. In a sample of adults above 50 years old, age proved significant in 5 of 8 subtests, however the authors suggest that these results should be expected, as it is known that visual ability decreases with age, affecting visual acuity, depth perception and object size among other things [30].

Various studies suggest the VOSP is a suitable test for detecting visuospatial impairment in mild AD patients [24,28,32]. A recent study showed that AD patient’s VOSP scores were consistently lower in comparison to non-AD patients, suggesting the VOSP may be suitable to screen for the presence of early AD [28,32]. The mean performance of scores on all of the VOSP subtests has also been shown to decline with advancement of dementia [24].

The administration of the VOSP begins with a preliminary visual sensory efficiency test, which is performed to determine that the patient has sufficient visual capacity to complete the other subtests. This is relevant because low visual acuity has been shown to have a meaningful negative impact on performance in the VOSP test battery [33]. This preliminary shape detection test requires patients to identify whether there is a degraded X on 20 patterned sheets of paper, with one point being given for each correct answer. Scores of 15 or lower effectively exclude participants from performing the VOSP battery. The full VOSP battery is comprised of four tests of object perception and four tests of space perception. If there are time constraints and the battery cannot be used in full, it is recommended that the silhouettes, progressive silhouettes and the cube Analysis be used as they are the most sensitive to the initial impairment of visuospatial function in AD [28]. A brief description of the tests can be found in Table 1, with cut off values for failure based on scores from the VOSP manual [34]. The VSOP manual is copyright protected material and is available at https://www.pearsonclinical.co.uk/Psychology/AdultCognitionNeuropsychologyandLanguage/AdultPerceptionandVisuomotorAbilities/VisualObjectandSpacePerceptionBattery(VOSP)/VisualObjectandSpacePerceptionBattery(VOSP).aspx

## 6. Age-Related Cataracts, Reduced Visual Acuity and Dementia

### 6.1. Cataracts

Cataracts are the primary cause of visual impairment worldwide, with an estimated prevalence of 30% in people aged 65 years or older in the UK [35]. The World Health Organization estimate that of the 45 million people worldwide with visual impairments that leave them unable to walk unaided, 46% have cataracts. Both cataracts and AD are primarily age-related diseases, and as such, their prevalence is expected to continue to increase over the coming years. Risk factors for cataracts include age, smoking, hypertension, diabetes and obesity among others [36,37]. A cataract is defined as an opacification of the lens of the eye that can cause a variety of visual impairments, including blurred or distorted vision and blindness in advanced cases. The pathological changes that define cataracts may present unilaterally or bilaterally, causing varying degrees of disability to individuals. Presenting symptoms of cataract are gradual painless loss of vision and lower visual acuity, glare when driving, monocular diplopia and difficulty in reading. Patients presenting with suspected cataracts undergo a comprehensive eye examination including refraction, IOP measurement, slit-lamp and dilated fundoscopy, in order to rule out differential diagnoses. Cataract removal by phacoemulsification and intra-ocular lens implantation is the surgical management of choice to improve vision.

### 6.2. Commonalities between Cataracts and AD

Age-related cataracts and AD share common risk factors and aetiologies. Cataracts and dementia are age-related degenerative conditions, and both may be accelerated by common risk factors. Given the commonality of risk factors, dementia patients may be expected to have higher incidence of cataract and vice versa. The risk factors for both AD and cataract overlap considerably, and include advancing age, female gender, smoking, diabetes, obesity, lower socioeconomic class (lower educational attainment) and vascular factors [36,38,39,40,41,42,43,44,45]. Both AD and cataracts may share a common fundamental mechanism and pathogenic pathway and molecular studies of AD patient’s cataract have shown accumulation of β-amyloid (Aβ) misfolded proteins bearing similarity with neural changes [46].

### 6.3. Cognitive Impairment and Reduced Visual Acuity

There is a strong association between cognitive test scores and visual acuity [47], with longitudinal data showing that decline in cognitive test scores and worsening visual acuity are significantly correlated [48]. The underlying mechanism of this correlation is unclear although multiple hypotheses have been proposed. The first (cognitive resource hypothesis) asserts that a decline in cognitive function leads to reduced visual ability and therefore subjects have an inability to see and interpret test material. Patients with lower visual acuity may therefore score more poorly on cognitive tests, falsely giving a diagnosis of cognitive impairment. This additional effort required for reading under these impoverished stimulus conditions is thought to consume cognitive resources needed for effective use of memory [49]. The second hypothesis (common factor theory) asserts that cognitive test scores are low due to a true cognitive impairment, and that a common factor underlies both the visual and cognitive decline present [50]. A third hypothesis is that a combined mechanism is responsible, and that both the previous hypotheses interact and each play a role [50].

## 7. Justification for Screening for AD by Eye Clinics

AD and cataracts are significant global health concerns, with both having significant financial health and social impacts for patients and those connected with them. As the population is aging and age is a significant risk factor for both diseases, both AD and cataracts are expected to increase in number worldwide.

As discussed, the benefits of early AD diagnosis are numerous and extend to patients, carers, healthcare professionals and society. Patients may benefit psychologically, be able to make informed decisions while they still have capacity and put into place interventions and lifestyle changes that maintain quality of life as much as possible. Carers are able to better plan support, while healthcare services are better able to plan their workload, allocate resources and anticipate future demand. For society, although the only data available is from economic modelling, this suggests that early diagnosis may effectively reduce the amount of time spent in care homes for AD patients, and therefore significantly reduce the fiscal demands of AD.

Patients with AD often present late in their disease, when they are no longer able to compensate for their cognitive decline, and carers are no longer able to cope. There is often an acute deterioration requiring assessment and respite due to carer burnout, as carers have often not been receiving support in their role or are even yet to recognise that this has become their role. In contrast, both patients and carers are likely to be more recognizant of declining vision and present to healthcare services when it begins to affect their day-to-day life. We propose that cataract clinics offer a viable and potentially cost-effective screening platform to identify early-stage AD during the patients’ post-surgery period. The VOSP takes an average of 12 min to administer, and opticians and ophthalmic nurses could be trained to do this. Awareness, in addition to further data arising from studies in this area will strengthen the argument for additional funding, although the additional funding required to administer the test is very modest and affordable.

As previously discussed, very mild AD patients have been shown to have visuospatial deficits and they are among the earliest manifestations of AD [23,28,51]. A recent study suggested that visuospatial abilities may decline in AD up to 3 years before clinical diagnosis, with a decline in global cognitive abilities following during the next year [52,53]. It has been suggested that this early visuospatial decline occurs as visuospatial function relies on parietal lobes that are damaged in early stage AD [23]. The MOCA (commonly used in General Practice) evaluates for a range of cognitive deficits (concentration, memory, language, etc.) including visuospatial deficits [54]. However, given the small size of the section of the test that evaluates this specifically (5 out of a possible 30 points), we hypothesise that it would be less likely to pick up subtle deficits in visuospatial function. In comparison, the VOSP provides a more robust analysis of a patient’s visuospatial abilities and has been shown to differentiate patients with early stage AD from controls, suggesting an increased likelihood of detecting early AD in comparison with non-specific visuospatial tests such as the MOCA [28]. The justification for utilising the VOSP in eye clinics is that the current tests administered by G. P’s may be less sensitive in detecting early AD.

## 8. Proposed Clinical Paradigm

We propose the VOSP test should be administered four to six weeks after surgery, as this is a common timeframe for recovery from surgery, and this is therefore the standard post-operative clinic follow up time in NHS treatment [55]. Cataract surgery has been shown to improve both visual acuity and contrast sensitivity [56,57,58]. Improved visual acuity and contrast sensitivity may optimise the VOSP test scores, with evidence supporting this proposal arising from studies that simulated reduced visual acuity in neuropsychological testing [33,59]. Hann et al. simulated cataracts by asking participants to wear glasses covered with blurred semi-transparent foil, and evidenced that this simulated low visual acuity had a meaningful negative impact on the performance in the VOSP and other tests that required visual input [33]. See et al. utilised cataract simulation goggles to reduce vision and contrast sensitivity, effectively replicating cataracts [59]. Although See et al. did not use the VOSP test specifically, comparable tests that required visual input such as letter matching and symbol recall evidenced lower scores in participants wearing these cataract simulation goggles. We suggest that the results of these studies may be extrapolated to suggest that the VOSP should be administered post-cataract surgery as the results from these studies [33,59] suggest that patients with low visual acuity may score poorly on neuropsychological tests due to biases caused by poor vision. Eliminating compounding factors such as cataracts may therefore offer a more optimised care pathway.

**Figure 1:**
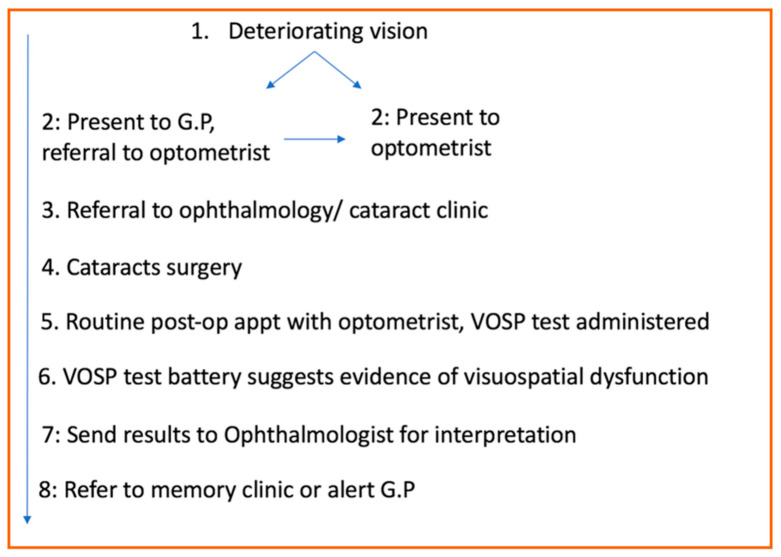
Proposed clinical paradigm.

When patients initially start to notice visual decline, they may present directly to their optometrist or to their GP. Optometrists perform ocular examinations, and if they diagnose a cataract the patient is referred to an ophthalmology department. Patients are then assessed by the ophthalmologists to determine the suitability for cataract surgery. Following surgery, the current treatment pathway offers patients routine follow appointments with the optometrist, who tests for visual acuity and evaluates how successful the surgery has been (Figure 1).

We propose that the VOSP can also be introduced at this stage by the optometrist to test for visuospatial deficits. Testing prior to surgery is not a practical option, as previously discussed any deficit in visual acuity is likely to obfuscate the VOSP test and subsequent score, which could potentially result in a false diagnosis of visuospatial difficulties when the problem may be only visual acuity. This risk can be obviated by testing after cataract surgery. The standard follow-up appointments with the optometrist are currently only to examine visual acuity, and as this alone is not a sufficient screening test for visuospatial deficits, a specific test such as the VOSP is needed. Administering the VOSP would traditionally be the domain of a neuropsychologist; however, the VOSP test battery requires minimal training on the part of the administrator and could be completed by a variety of healthcare professionals, including optometrists. Scores can be sent back to the ophthalmologist or to the primary care physicians who in turn can make an appropriate action and plan a care pathway. This may increase the likelihood of an early diagnosis and introduction of preventive measures not only will improve the quality of the life of the affected individuals but also could reduce the social and financial burden of AD.

## 9. Conclusions

The role of eye clinics in screening for AD is an evolving concept, and more research is needed to illuminate and assess the most appropriate pathways to facilitate early diagnosis. An early diagnosis of AD may be beneficial to patients, caregivers, healthcare professionals, and to society as a whole by reducing the financial burden arising from severe dementia. As early presentation and treatment of cataract surgery is becoming the norm and AD and cataract patients share overlapping risk factors, we propose that offering AD screening during post-operative follow up assessment for cataract surgery may offer an opportunity to screen for early AD.

## Figures and Tables

**Table 1:** A brief description of the tests with cut off values for failure based on scores from the VOSP manual.

**Object Perception**	
Incomplete letters	Incomplete letters are shown to the patient, and he is asked to name or identify them. Cut off value for failure: 16/20
Silhouettes	Silhouettes of animals and objects are shown to the patient, and he is asked to identify them. Cut off value for failure: 15/30
Object Decision	Boards with four stimuli are presented. Only one of these stimuli represents something real; the other forms are not defined (distractor stimuli). The patient is asked to identify and name the stimulus that represents the real shape. Cut off value for failure: 14/20
Progressive Silhouettes	This test consists of two series in which boards depicting an object are presented; 10 boards depict a gun, and 10 boards show a trumpet. The first board shows the silhouette of the object, and each successive board shows a more complete picture of the object. The patient is asked to identify these two objects with the smallest possible number of boards. The number of stimuli needed to identify these objects is recorded. Cut off value for failure: 15/20
**Space Perception**	
Dot count	The patient is asked to count how many black dots there are on a white card. Cut off value for failure: 8/10
Position discrimination	Each board has two squares with a black dot in the centre each. In one of the squares, the point is exactly in the centre, while the other point is slightly off-centre. The patient is asked to identify in which square the black spot is located exactly in the centre. Cut off value for failure: 18/20
Number location	Each board has two squares arranged one above the other. The top square contains numbers arranged randomly. The bottom square contains only a black dot. The patient is asked to identify which number corresponds to the black dot. Cut off value for failure: 07/10
Cube Analysis	Each board features the design of solid structures. The patient is asked to identify how many solids (cubes) there are on each board. Cut off value for failure: 06/10
